# Hydatid cyst of the pericardium: a case report

**DOI:** 10.11604/pamj.2014.19.330.5542

**Published:** 2014-11-27

**Authors:** Abdelilah Mouhsine, Ahmed Belkouch, El Mehdi Athmane, Redouane Roukhssi, Abdelghani El Fikri, Lahcen Belyamani, Mbarek Mahfoudi

**Affiliations:** 1 Department of Radiology, Avicenna Military Hospital, Faculty of Medicine and Pharmacy, Marrakech, Morocco; 2 Emergency Department, Mohamed V Military Hospital of Instruction, Faculty of Medicine and Pharmacy, Rabat, Morocco

**Keywords:** CT scan, echocardiography, hydatid cyst, MRI, pericardium

## Abstract

Pericardial hydatid cystis a rare condition; its clinical presentation is variable. It can reveal straightaway at the stage of life threatening complications. We report the case of a 17 years old female Arab patient, who complained of a sudden onset dyspnea, clinical examination was poor; the diagnosis was suspected by echocardiography and confirmed by the CT scan and hydatid serology. Furthermore, no other location was noted. Surgical treatmentwas proposed. The modern cross-sectional imaging especially CT scan and MRI revolutionized the diagnosis of this rare hydatid location.

## Introduction

Hydatidosis rages at the endemic state in some regions of the world, it is a major public health problem; it is due to the human´s accidental infestation by the larval form of the parasite [1]. The hydatid cyst of the pericardium is a rare localization; it is a serious condition because it exposes to life threatening complications.

## Patient and observation

**Figure 1:**
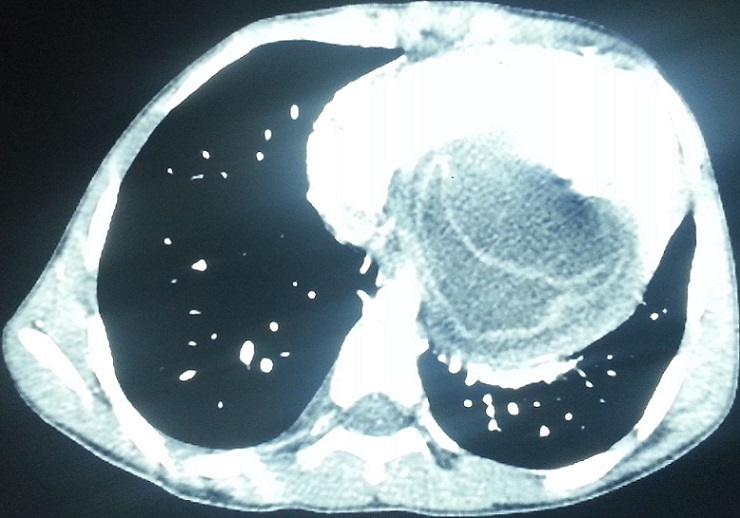
Thoracic helical CT scan in axial acquisition after injection of contrast material showing a pericardial cystic lesion next to right cavities especially the right ventricle, a detached layer is seen within the lesion, which compresses the ventricular myocardium

**Figure 2:**
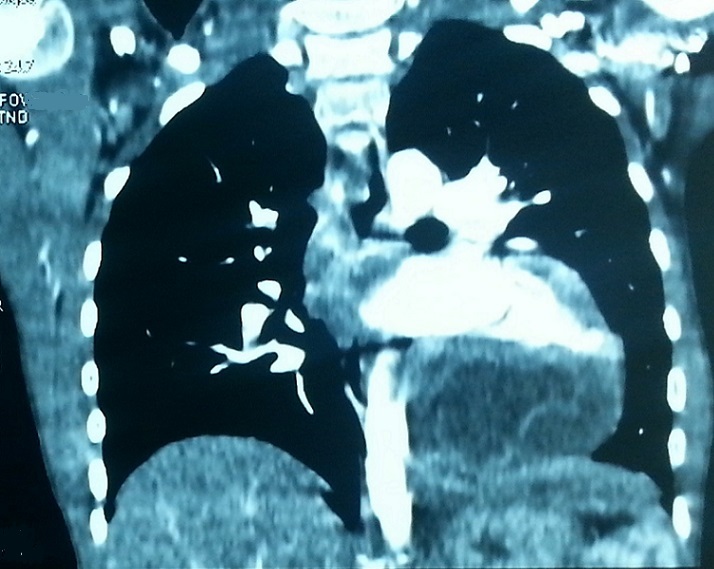
Reconstructed coronal CT scan showing the same image of the pericardial cystic lesion next to the right ventricle

**Figure 3:**
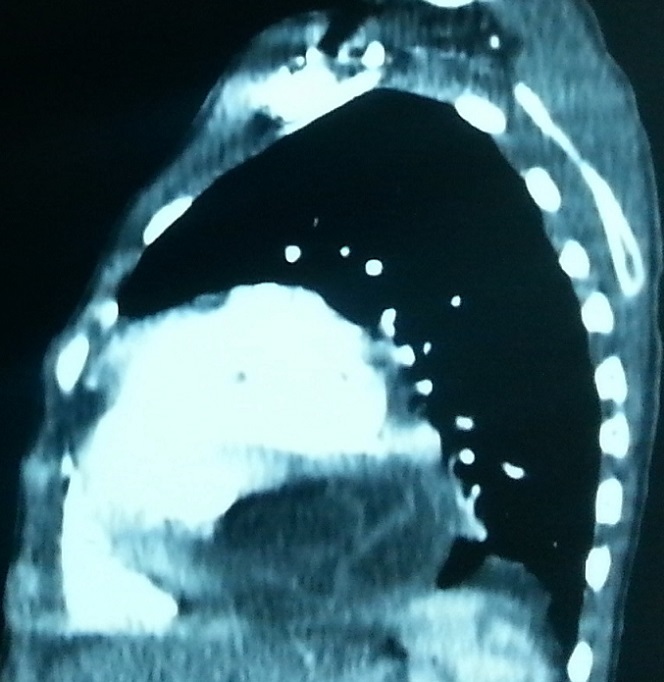
Sagittal CT scan reconstructionsafterinjectionof contrast materialinmediastinalviewshowing the same lesionnext to right cavitieswith a detached layer within it

**Figure 4:**
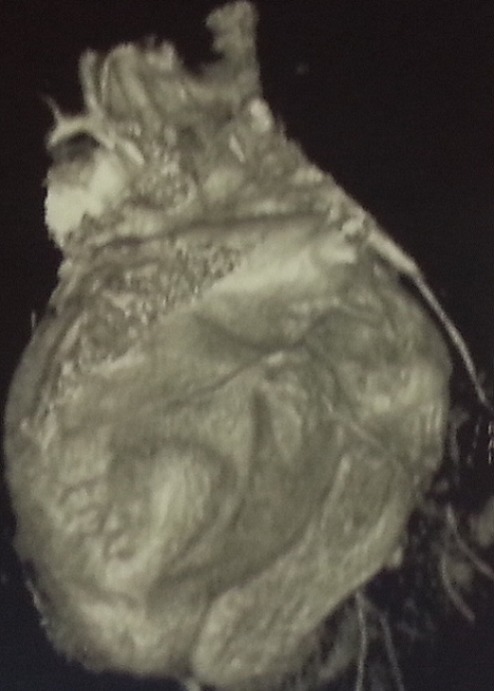
Pericardial lesion next to right cavities

A 17 years old young woman was admitted to the emergency department for the management of a sudden onset dyspnea. She had no history. Clinical examination revealed a dyspneic but conscious patient, with no fever. Respiratory rate was 22 cycles/min, SpO2=92% in the open air, heart rate=114 beats/min, blood pressure=144/95. She received supplemental oxygen at 4 l/min via nasal cannula, SpO2 improved and increased to 98%. A biological assessment was requested and revealed anemia to 10g/dl of hemoglobin. The remaining biological record was normal. Chest radiograph showed discrete cardiomegaly, cardio thoracic index was at 0.55. No other pleural or pericardial abnormalities were noted. Transthoracic echocardiography showed a fluid compartmentalized cystic anechoic lesion, homogeneous, compressing the right cavities of the heart. Chest CT scan (Figure 1, Figure 2, Figure 3, Figure 4) showed discrete cardiomegaly with pericardial lesion facing the right cavities especially the right ventricle, this lesion is relatively toned, fluid density, encapsulated, seat of few banded membrane structures. This lesion measures 8 cm (longitudinal axis), 7 cm (transverse axis), and compresses the next ventricular myocardium, it suggests first of all pericardial hydatid cyst. Abdominal ultra sound was normal. There after the patient was admitted to cardiology; hydatid serology was performed and was positive. Surgical treatment was proposed but the patient refused it.

## Discussion

Hydatidosis is a problem of public health in some regions of the world where it rages at the endemic state, as north-Africa, some regions of the Mediterranean Sea and in the Middle East [1]. It is a parasitosis due to the infestation by the larva of the tenia echinococcus granulosis. Humans are accidental hosts of this parasite after ingestion of the parasite eggs or scolex. These eggs cross the small intestines after having lost their envelopes, join the portal circulation and then reach the liver. Hydatid cyst affects in most cases the liver (59-75%) the lungs (27%) kidneys (3%) bones (1-4%) the central nervous system (1-2%). The heart, spleen, pancreas, muscles, adrenal glands, the parotid gland, thyroid and prostate are exceptional locations of this parasite [2]. Cardia c location represents 0.5 to 2% of all hydatid cyst localizations [3]. It is isolated in 2/3 of time. The intracardiac location varies: left ventricle is involved in 60% of cases, interventricular septum in 9-20%, right ventricle and right atrium in 4-17%. Pericardial hydatid cyst has been very little reported in the literature, it is a serious condition because it exposes to severe complications [4]. The clinical manifestations of pericardial hydatid cyst depend of its seat, size and number of cysts [2, 5]. The symptoms may be non-specific as chest pain, palpitation, effort dyspnea as for our patient, sometimes the discovery is fortuitous; it can also manifest as complication: intrapericardial rupture causing tamponade, cardiac arrest. Chest radiography is not contributive to the diagnosis, it may show cardiomegaly with uni or bilobed deformation of the cardiac silhouette, arcuate calcifications, and can be associated with pulmonary localization. In our case it revealed a discrete isolated cardiomegaly.

Transthoracic or transesophageal echocardiography is an indispensable and efficient examination, it can analyze the cyst characteristics, uni or multi locular, its topography, appearance and its relationship with the heart chambers. It may reveal an associated pericardial or pleural effusion. Some aspects are strongly suggestive of hydatid cyst as a detached layer or a multivesicular appearance [6]. For our patiente chocardiography revealed the lesion but it was the CT scan that permitted to specify its characteristics. Indeed CT scan better specifies its relationship with adjacent vital structures, it is usually a hypodense Uni-or multicelled lesion, not enhanced after injection of contrast material unless infection; it also allows to search other local and loco regional lesions. Abdominal ultrasound is useful to search for an associated hepatic localization. MRI is indicated in case of doubtful diagnosis or in case of divergence between the echocardiographic and CT scan findings. It permits a better characterization of the lesion and precise better the anatomical relationships. The hydatid cyst presents then as a liquid signal lesion in low signal T1, and high signal T2, daughter cysts are revealed in low signal T1 and in low or T2 high signal according to the presence or not of scolices achieving the aspect of the “cyst in the cyst”. The detached layers within the cyst are responsible of a linear or banded low signal inside the cyst giving the aspect of the" Rim sign". Hydatid serology is not very useful to the diagnosis. It is only positive in half cases. Immuno-electrophoresis is more specific. The pathological examination confirms the diagnosis. The treatment is surgical [7, 8], although some studies have reported cases treated using benzimidazoles (albendazole, mebendazole) [9].

## Conclusion

The pericardial location o fhydatid cyst is rare, its clinical expression is polymorphic and can expose to life threatning complications. The cross-sectional imaging is the gold standard for the diagnosis especially CT and MRI. Serology may be helpful. Treatment is primarily surgical.
